# Immune Thrombocytopenia and Cerebral Venous Sinus Thrombosis Following COVID-19 Vaccination: A Case Report

**DOI:** 10.7759/cureus.34272

**Published:** 2023-01-27

**Authors:** Saurabh Kataria, Rezaur Rahman Reza, Adesola A Agboola, Khalid H Mohamed, Alaa S Mohamed, Nimra Zahid, Muhammad Haseeb, Hira Nasir

**Affiliations:** 1 Neurology, Ochsner Louisiana State University Health Sciences Center, Shreveport, USA; 2 Clinical Observation and Research - Neurology and Neurocritical Care, University of Missouri Health Care, Columbia, USA; 3 Distant Research - Neurology, West Virginia University, Morgantown, USA; 4 Neurology, Jalalabad Ragib Rabeya Medical College, Sylhet, BGD; 5 Pathology and Laboratory Medicine, Dele Hospitals, Lagos, NGA; 6 Neurology, Sheffield Teaching Hospitals NHS Foundation Trust, Sheffield, GBR; 7 Neurology, Augusta University, Augusta, USA; 8 Internal Medicine, Allama Iqbal Medical College, Lahore, PAK; 9 Internal Medicine, Mount Sinai Hospital, Brooklyn, USA; 10 Internal Medicine, Mayo Hospital, Lahore, PAK

**Keywords:** covid-2019, cerebral venous sinus thrombosis (cvst), vaccine-induced prothrombotic immune thrombocytopenia, vaccine-induced thrombocytopenia, covid associated thrombocytopenia

## Abstract

Mass vaccination against coronavirus disease 2019 (COVID-19) has been safe and effective. The ongoing emergence of vaccine-induced complications has challenged the public trust in vaccination programs and, though uncommon, can lead to significant morbidity and mortality. Vaccine-induced immune thrombocytopenia and thrombosis (VITT) is a rare and fatal complication of the COVID-19 vaccine. We present a rare case of VITT in a young female who presented with worsening headache, body rash with deteriorating neurological deficit after 12 days of the second dose of the ChAdOx1 COVID-19 vaccine. Initial blood tests showed thrombocytopenia with deranged clotting time and D-dimer levels. Her computed tomography venogram showed thrombosis in the left transverse sinus, and she was diagnosed with a provisional diagnosis of VITT. She initially managed with dexamethasone, intravenous immunoglobulins, and apixaban to reverse the autoimmune process. Our case highlights the clinical course, diagnosis, and management of VITT, which will assist physicians in the timely recognition and adequate management of VITT.

## Introduction

Vaccine-induced immune thrombocytopenia and thrombosis (VITT) is an uncommon but fatal complication following vaccination against coronavirus 2019 (COVID-19) [1]. VITT is also called vaccine-induced prothrombotic immune thrombocytopenia (VIPIT) or thrombosis with thrombocytopenia syndrome (TTS) [2]. VITT manifests with sudden onset worsening headache, which may be associated with bleeding manifestations followed by neurological manifestation grossly and altered sensorium. Clinical manifestation primarily depends on the involved vessel and may involve deep vein, acute arterial thrombosis, pulmonary embolism, and rarely cerebral and splanchnic veins [3]. The estimated risk for VITT is at least 1:50,000 for patients under 50 and 1:100,000 in populations over 50; however, the risk is amplified in younger people and recipients with the first dose of vaccine [4]. VITT has also been reported following vaccines against other viruses such as measles, mumps, rubella, human papillomavirus, hepatitis B, and poliovirus [5]. Herein, we report a rare case of VITT in a young patient following the COVID-19 vaccine.

## Case presentation

A 28-year-old female was brought to the emergency department with complaints of severe headache and generalized fatigue for the last week. Headache was gradual in onset, generalized, and progressive, with no aggravating and relieving factors. She also complained of walking difficulty and weakness in the right half of her body over the last night. She had no previous medical history or family history of the thrombotic event. She smoked for five years, quit smoking three years ago, and had no history of alcohol and illicit drug use, and she had not been using any hormone replacement therapy or contraceptive pills. She received her second dose of the ChAdOx1 COVID-19 vaccine 12 days ago.

On examination, she was anxious, afebrile, well-oriented in time, place, and person, and hemodynamically stable. There were no signs of meningeal irritation. A generalized petechial rash on her arms and legs was observed. On neurological examination, she could not walk, with decreased power, grip, and sensations on the right side of the body. The rest of the systemic examination was unremarkable. Initial laboratory evaluations were remarkable for thrombocytopenia and elevated D-dimers (Table 1).

**Table 1:** The results of initial laboratory investigations INR: international normalized ratio, APTR: activated partial thromboplastin ratio

Parameter	Day 1	Day 2	Day 3	Reference value
Hemoglobin	12.4	12.1	11.8	12.1-15.1 g/L
Red cell count	4.31	4.22	4.10	4.20-5.65 million cells/uL
White cell count	5.5	5.2	4.9	4000-11000/mm^3^
Platelet count	21,000	24,000	37,000	150,000-350,000/mm^3^
Lactate dehydrogenase	282	279	270	140-289 IU/L
Prothrombin time	13	14.6	12.9	10-11.7 seconds
APTR	1.11	1.4	1.2	0.85-1.10
D-dimers	>5000	>5000	>5000	< 0.50 ng/dl
INR	1.5	1.4	1.4	0.8-1.2
Fibrinogen level	1.4	1.6	1.4	1.8-3.6 g/L

**Figure 1:**
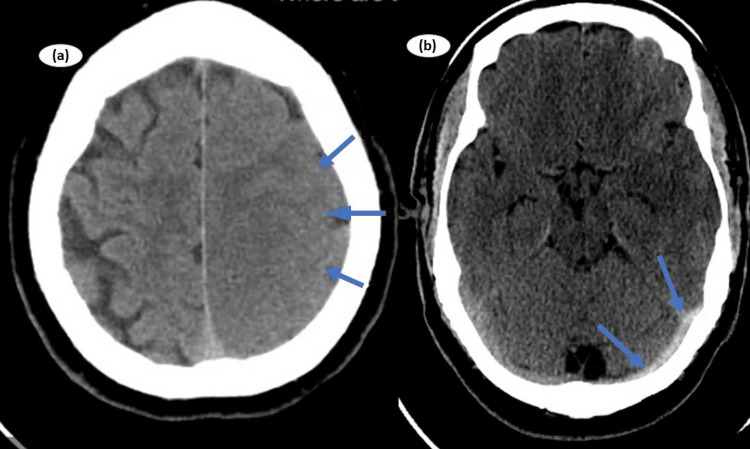
Computed tomography showing vasogenic edema of left cerebral hemisphere (a) and hyperdense left transverse sinus (b)

**Figure 2:**
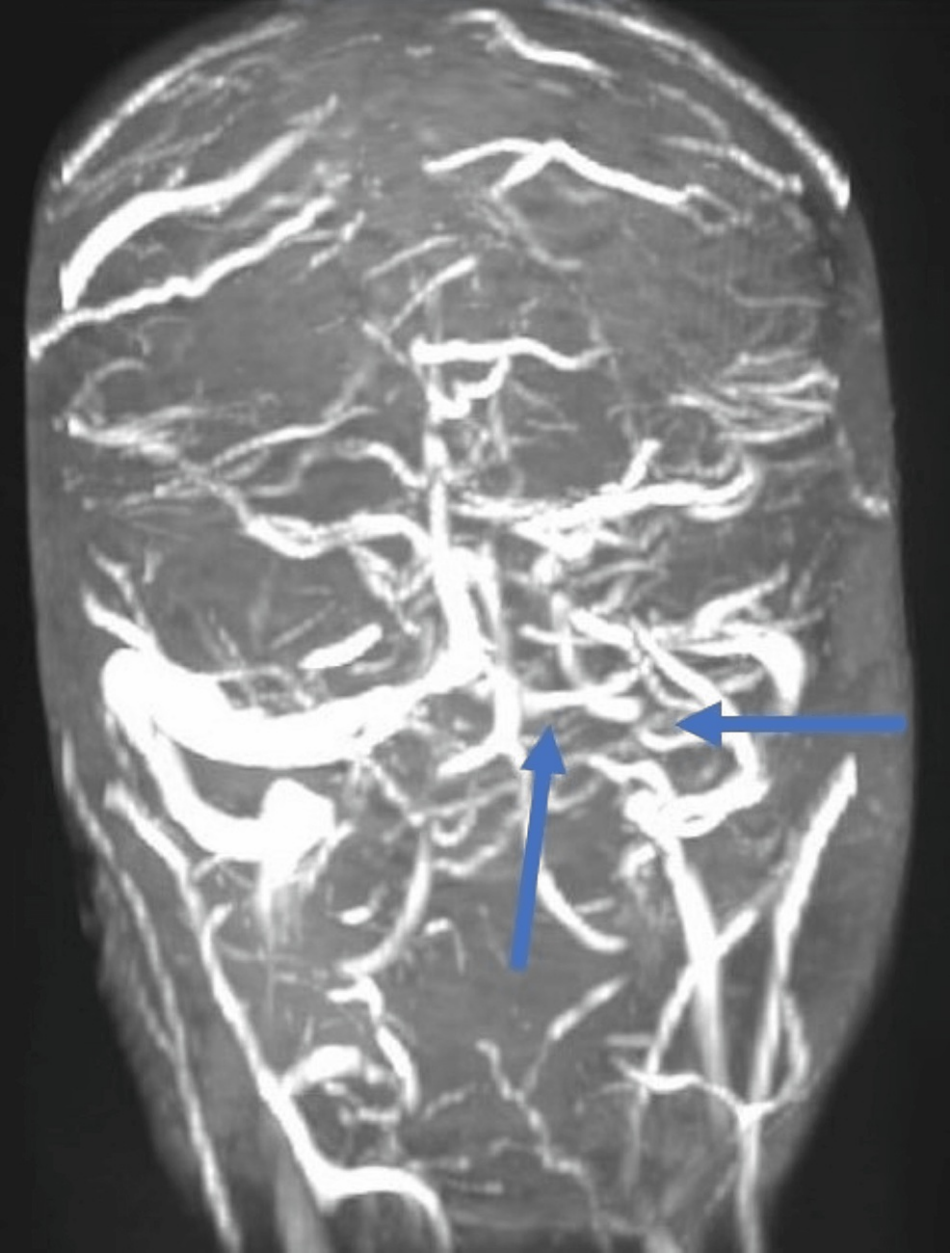
CT venography showing a filling defect in the left transverse sinus (arrows showing the absence of the left transverse sinus)

She underwent computed tomography (CT), which revealed cerebral vasogenic edema on the left side of the cerebral hemisphere and hyperdense left transverse sinus (Figure 1). CT venography was performed, which revealed a filling defect left transverse sinus (Figure 2). She was screened for antiphospholipid antibody test, autoantibodies, paroxysmal nocturnal hemoglobinuria, direct antiglobulin test, ADAMST13 (a disintegrin and metalloproteinase with thrombospondin type 1 motif, member 13), and platelet factor 4 (PF4) antibodies, which were negative except for PF4 antibodies (>1.0 IU/ml). Based on her clinical, biochemical, and radiological picture, a provisional diagnosis of VITT was made. She was managed with high-dose intravenous dexamethasone 40 mg daily. She was also commenced on intravenous immunoglobulin (IVIG) at a dose of 1 g/kg daily to reverse the autoimmunity for two days. In consultation with a hematologist, she was started on a low amount of apixaban (2.5 mg) twice daily because of the perceived increased risk of bleeding.

She was observed closely, and her clinical symptoms gradually improved over 10 days. She was self-ambulatory on discharge with a near-complete resolution of neurological manifestations after two weeks. She remained on apixaban with a normal hemogram on regular follow-up and was continued on anticoagulation for six months.

## Discussion

Mass vaccination program against COVID-19 infection is actively ongoing worldwide, and global vaccination has led to control of the COVID-19 pandemic. Safe and effective vaccination against COVID-19 remains the mainstay of control of coronavirus because of the non-availability of universally recommended treatment, leading to vaccine hesitancy and resistance due to fear of safety concerns and serious adverse events. The vaccines against COVID-19 have different mechanisms of action, leading to different injection procedures, dosages, and timing [6]. Most vaccines are well-tolerated and have shown promising efficacy and immunogenicity; however, vaccine-associated side effects and adverse events have also been reported. Headache, myalgia, malaise, and injection site reactions are commonly reported side effects of the COVID-19 vaccine. Vaccine-associated adverse events can be classified into gastrointestinal, cardiovascular, hematological, neurological, and immunological [7].

Among hematological and immunological complications, patients have also been reported with thrombosis, coagulation disorders, thrombocytopenia, abnormalities in fibrinogen levels, D-dimer count, or partial thromboplastin time [8]. VITT, a life-threatening emergency, has also been underlined in the literature, although not widely reported. Butler-Manuel W et al. reported a case of VITT and severe immune thrombocytopenia after 11 days of the COVID-19 vaccine [9]. Sivaramakrishnan P et al. also highlighted a case of VITT in a middle-aged woman following one month of the COVID-19 vaccine and responded to steroid therapy [10]. Mehta PR et al. described two cases of severe thrombocytopenia with fatal VITT following the first dose of the COVID-19 vaccine. Both patients presented with severe headaches and progressive neurological deterioration [11]. An analysis of 25 studies highlighted that 31 out of 80 patients who developed thrombosis died, and mortality was dependent on the onset of symptoms following vaccination and independent of vaccine type, platelet count, and comorbidities [1,4].

The pathophysiology of VITT needs to be clearly defined. It is hypothesized that vaccines induce the immune-mediated response, and immune complexes are performed like heparin-induced thrombocytopenia (HIT). Postvaccination antibodies against platelet antigens trigger massive platelet activation against PF4, leading to ITT. Cross-reactivity between PF4, platelets, and vaccine is considered a potential contributing factor in the pathogenesis of VITT, leading to platelet activation and thrombosis [4,5,12].

Recommended management includes consultation from a hematologist as soon as possible. Systemic glucocorticoids and IVIG 1 g/kg body weight are recommended as first-line management. Non-heparin-based anticoagulation should be commenced if there is a high suspicion of VITT diagnosis and the results of PF4 serology are awaited. In the case of thrombocytopenia, platelet transfusions should be avoided unless the platelet count is above 30,000 and there is no active bleeding. The fibrinogen level must be corrected to maintain a fibrinogen level of >1.5 g/L, and all heparin-based flushes must be avoided until VITT is ruled out. All the patients diagnosed with VITT are advised to continue systemic anticoagulation for a minimum of three months and periodic follow-ups [2,3,13].

## Conclusions

Although mass vaccination against COVID-19 has been effective, immunological complications, mainly VITT, have been reported following vaccination. VITT is a serious and rare life-threatening adverse event, and our case highlights the importance of the timely recognition and management of post-vaccine complications. Despite high mortality and more augments in favor of the causal relationship between the COVID-19 vaccine and VITT, timely diagnosis and management can be lifesaving.
